# Exacerbating the Tragedy of the Commons: Private Inefficient Outcomes and Peer Effect in Experimental Games with Fishing Communities

**DOI:** 10.1371/journal.pone.0148403

**Published:** 2016-02-10

**Authors:** Jorge Higinio Maldonado, Rocío del Pilar Moreno-Sanchez

**Affiliations:** 1 Department of Economics – CEDE, Universidad de los Andes, Bogotá, Colombia; 2 Conservation Strategy Fund, Bogotá, Colombia; Tianjin University of Technology, CHINA

## Abstract

Economic Experimental Games have shown that individuals make decisions that deviate down from the suboptimal Nash equilibrium. However, few studies have analyzed the case when deviation is above the Nash equilibrium. Extracting from above the Nash equilibrium is inefficient not only socially but also privately and it would exacerbate the tragedy of the commons. That would be the case of a race to the fish when stocks are becoming depleted or driver behavior on a highly congested road. The objective of this study is to analyze private inefficient extraction behavior in experimental games and to associate the type of player and the type of player group with such inefficient outcomes. To do this, we carried out economic experimental games with local coastal fishermen in Colombia, using a setting where the scarcity of the resource allows for an interior Nash equilibrium and inefficient over-extraction is possible. The state of the resource, the type of player and the composition of the group explain, in part, this inefficient behavior.

## Introduction

Conflicts associated with common-pool resources (CPR) have been widely studied in the economic literature, including in the fields of game theory and behavioral and experimental economics. Social dilemmas have also been studied under the framework of evolutionary game theory; specifically, the evolution of cooperation in social dilemmas has been analyzed using methods of statistical physics that allow the incorporation, through simulations, of several variables present in real world systems, such as heterogeneity, agent mobility, utility interdependence and interaction between networks, among others [1–4].

Fisheries have been identified as the typical case of common-pool resources (CPR), whereby the impossibility of exclusion and rivalry between users result in their degradation. Gordon argued that this class of resources which is considered “free” would not be extracted at the proper time because for fishermen, fish remaining in the sea are valueless, inasmuch as there is no guarantee of finding them in the future if they are left behind today [5]. Hardin coined the expression “the tragedy of the commons” to describe the overuse and consequent depletion and exhaustion suffered by CPRs as a result of resource users’ individualistic behavior [6].

The tragedy of the commons has been formalized using non-cooperative game theory (NCGT), wherein communication between players is not permitted and all players have complete information about the payoffs associated with their respective decisions [7].

Predictions derived from non-cooperative game theory establish that under a CPR scenario, players selecting their best individual strategies will not reach a Pareto-optimal outcome, and that individual rational decisions will lead to outcomes that are collectively irrational, a paradox known as the prisoner’s dilemma [7]. In other words, individuals facing CPR dilemmas will make decisions that lead to a suboptimal Nash equilibrium, rather than pursuing strategies that would lead to a social optimum [8].

Evidence from economic experimental games has challenged this theoretical prediction, showing that individuals deviate down from the Nash equilibrium towards the social optimum [9], and make extraction decisions that balance both their own and collective interests [10–12], even when they are not allowed to make agreements [13, 14].

Games dealing with CPR are usually designed in such a way that the Nash equilibrium is a corner solution (i.e. the maximum allowable amount to be extracted). There are, however, some cases when the Nash equilibrium is an interior solution of the game and the players have the possibility of extracting even more than the Nash equilibrium. If so, players would be considered inefficient both from a social and a private standpoint and, as such, they would be exacerbating the tragedy of the commons.

In real situations, this could be possible when the resource is highly overexploited and scarcity is strongly affecting all users’ decisions, promoting an increase in rivalry over the good. In fisheries, it would be the case of depleted stocks where fishers engage in a race to the fish and extract excessively before the final exhaustion of the resource, even if this implies an even shorter lifespan of that resource. In a highly congested road, for example, at peak hours commuting car drivers are not willing to share the road or let other vehicles use the road space correctly, exacerbating the tragedy associated with the overuse of that road space, even if this is highly inefficient.

Despite the abundant literature regarding CPRs, few studies have researched the phenomenon of private inefficient outcomes: excessive over-extraction and exacerbation of the tragedy of the commons.

In an attempt to contribute to the understanding of those issues, the objective of this paper is to analyze the inefficient extraction behavior in experimental games and to associate the type of player to such inefficient outcomes. To do this, we used experimental economic games where scarcity of the resource makes the Nash equilibrium shift from a corner to an interior solution, and the players are allowed to extract even more than the Nash equilibrium.

The paper is organized as follows: complementing this introduction, we discuss the literature related to our research problem and present our contribution and hypotheses. The second section describes the methods used for answering the research question, and, in the third, we present and discuss the main results. Finally, in the last section, some policy implications and conclusions are examined.

### Background

Private inefficient outcomes in experimental games have been found and analyzed by some authors in both static and dynamic frameworks. In CPR games the Nash equilibrium determines the private efficient level of extraction. Deviations below the Nash equilibrium may reflect collective behavior or others-regarding preferences, as individuals may incorporate a consideration of collective interests in their individual extraction decisions. That is, individuals do not necessarily pursue purely self-interested strategies, as predicted by theory [15]. Conversely, when individuals extract more units than those predicted by the Nash strategy and so the deviation is above the Nash equilibrium, the conclusion is that they are being highly inefficient, inasmuch as they are making decisions that negatively impact their own private returns.

In general, the literature on experimental games tends to focus more on analyzing individual deviations towards socially efficient outcomes than privately inefficient ones. Sometimes, privately inefficient outcomes in CPR games have been seen as outliers of the experiment that have not been further analyzed [15].

However, some researchers have associated this inefficiency in CPR games with an intensification of the tragedy of commons. For instance, Corners and Sandler, using a static framework, analyzed the role of non-zero conjectural variations on the behavior of fishermen [16]. These authors argue that the conjectures of an exploiter with respect to the way in which other exploiters will respond to its own extraction effort are absent in CPR models. Specifically, they establish that if non-zero conjectural variations are incorporated, individual responses will deviate either negatively or positively from the Nash equilibrium. As a result, if conjectures are negative the optimal fleet size will be greater than the Nash prediction and consequently “the tragedy of the commons will be intensified” [16].

Dynamic effects might also exacerbate CPR-related problems, as individuals might not consider the full impact of their own and others’ current decisions on future extraction costs. Herr, Gardner & Walker used laboratory experiments to analyze time-independent and time-dependent externalities of non-renewable commons, finding not only that the myopic behavior of individuals exacerbates CPR-related problems, but also that even those individuals who take into account the current and future effects of extraction decisions will likely enter into a race for resources if they believe others are acting myopically [17].

Other studies have analyzed the private inefficiency associated with the under-contribution of individuals in public-good games. In linear public-good games, the maximizing private benefit strategy (i.e., the Nash equilibrium) is to allocate zero units to the public good and all units to private activity. However, the findings from experimental games contradict these predictions, as individuals tend to make important contributions to the public good. This finding is robust for treatments where linear game designs do not allow for negative contributions. To analyze the possibility of under-contribution in public game experiments (i.e. deviations below the Nash equilibrium), some authors have modified the payoff structure to allow for interior solutions, or partial contributions, thus defining payoff functions that are non-linear with respect to both private and public goods [18–21]. The findings from such studies have been ambiguous; some have found that over-contribution is not significant for high levels of equilibrium contribution, and that individuals actually tend to under-contribute [20], while others report that over-contribution is significant [18, 19].

In public goods games, the literature appears to support the idea that the level of the predicted equilibrium contribution plays an important role with respect to contribution decisions, and affects the existence of under-contribution as well as its magnitude. For instance, Willinger and Ziegelmeyer [21] analyzed the strength of the social dilemma vis-à-vis contribution behavior. They reduced the strength of the social dilemma by moving the equilibrium contribution towards the social optimum, and found that over-contribution is only significant at a low level of equilibrium contribution. This confirms Isaac and Walker’s findings, which show that average over-contribution is reduced when the equilibrium level moves towards the Pareto optimum [20].

Despite the similarity of their findings regarding over-contribution to those of Willinger and Ziegelmeyer, Isaac and Walker found that subjects do tend to under-contribute when faced with high levels of equilibrium contribution: at the two highest levels of Nash equilibriums, average investments in the public good are below the Nash prediction [20]. Isaac and Walker also found that upward and downward biases are not the result of pure error. In sum, Isaac and Walker’s paper shows that “within the same experimental group, some individuals follow investment strategies that are highly ‘cooperative’ while others follow strong ‘free riding’ strategies, which might explain the under contribution observed in the treatments with highest predicted equilibrium levels.”

Inefficient outcomes could be related with a particular “type” of individual. Some approaches have classified the players according to their responses. Ostrom [22] shows that from a pool of players in a repeated setting, several reciprocity norms can be found: 1) always cooperate first, 2) cooperate if others are trustworthy, 3) cooperate if others have already cooperated, 4) never cooperate, 5) mimic (1) or (2) but stop if free-riding is possible, 6) always cooperate. Another approach is the one proposed by van Soest and Vyrastekova [23], who propose that participants in games can be classified as individualistic (they maximize their pay-offs), competitive (they obtain gains from the game but not as much as individualistic players), and cooperative (also obtain less gains than individualistic participants). These studies, however, do not contemplate the possibility of inefficient behavior associated with over-extraction beyond the Nash equilibrium in CPR games.

Given previous approaches in examining social and private inefficiency in the use of public goods and CPRs, we focused our contribution on testing the following hypotheses: in dilemmas associated with CPR, specifically fisheries, individuals facing an abundance of resources tend to cooperate (that is, under-extract), even when no rules are applied. However, cooperation is reduced and individuals might even be privately inefficient when faced with resource scarcity, as they adopt a “race to the bottom for extraction-profit” strategy. This hypothesis could be presented in other terms: the social dilemma associated with the use of a CPR becomes weaker as the private maximizing-solution level moves towards that of the social (Pareto optimal) solution, whereby a lower level for the Nash equilibrium results from changes in the stock of the resource. We hypothesize, moreover, that this behavior is related with the type of player and the composition of the group to which each player belongs.

## Methods

### Theoretical model

To accomplish our objective, we adopted the dynamic model of profit maximization postulated by Moreno-Sanchez and Maldonado [24], which both captures the social dilemma of common-pool resources, and incorporates the inter-temporal effects of aggregated extraction. The model is based on an individual fisherman benefit function that is non-linear in both the level of private extraction (*x*) and the level of resource stock (*S*). The benefits (and costs) that individuals obtain from the extraction activity are in turn divided into two parts: i) the private benefit *f(*.*)*, which depends on the level of extraction (*x*), and the costs of which depend on the availability of the resource (*S*); and ii) the collective benefits or costs *g(*.*)*, resulting from the extraction decisions made by all of the fishermen using the resource which will affect its availability for other fishermen. This benefit function represents the profits from a common-pool resource (CPR) characterized by non-exclusion and rivalry:

$$\pi_{i,t}\;=\;f\left(x_{i,t},S_{t}\right)\;+\;g\left(\Sigma_{i}x_{i,t}\right),$$ (1)

where π_*i*,*t*_ indicates the benefits fisherman *i* obtains during period *t* from extracting the resource. The private portion of benefits *f(*.*)* is assumed to be a quadratic function of extraction (in order to capture the decreasing marginal benefits of extracting), and non-linear for the stock of the resource, with the assumption of a reserve-dependent cost (i.e., that the cost increases with a reduction in the stock, though not linearly). The collective portion of the benefit, function *g(*.*)*, is assumed to be linear for the level of extraction, and represents the effect of joint extraction on individual benefits. That is, the externality incurred on each user of the resource by the aggregated extraction [24].

The resource stock changes according to an evolution equation where the amount of the resource in period *t+1* will equal the stock at the beginning of period *t*, minus the extraction by all fishermen during that period, plus the net growth of the resource.

The Nash equilibrium of this model is estimated, for a set of specific functions by Moreno-Sanchez & Maldonado, and shows that the optimum private extraction depends positively on the available stock and on the marginal benefit of the resource (the price) and negatively on the extraction costs, the impact of aggregated extraction (the externality), and the discounted inter-temporal price of the stock of the resource.

To obtain the level of extraction maximizing social welfare, a central planner would aggregate the benefits of all the individuals. This equilibrium is also calculated by Moreno-Sanchez and Maldonado and shows that the social level of extraction must be lower than the privately efficient level of extraction [24].

This model therefore shows that private extraction decisions should differ from social optimum decisions and, moreover, that they can range across an ample spectrum depending on the value of the parameters and particularly on the level of stock. Lower resource levels should lead to lower levels of extraction as an efficient private decision, but there are still no constraints on moving up or down from this theoretically optimal level of extraction. Making decisions down the Nash equilibrium (closer to the social optimum) is commonly observed in experimental games, and can be associated to pro-social attitudes of players. Making a decision above the Nash equilibrium would imply not only an inefficient outcome from the social perspective, but also from the private perspective and would end up exacerbating the tragedy of the commons.

### Empirical Model

#### The model simulation and pay-off structure

In order to construct a pay-off structure that recreates the conflict between the collective and private interests, Moreno-Sanchez and Maldonado assign specific values to the parameters and determine the range of plausible extractions from one to eight units, following previous field experiments conducted by Cardenas [12]. Even though the dynamic model proposed by Moreno-Sanchez and Maldonado could imply several stocks of the resource and therefore many Nash equilibriums, in order to make the game practical, easy and understandable for real fishermen, simulated solutions are used for only two levels of stock: high (one of abundance) and low (one of scarcity). Correspondingly, it was only necessary to construct two payoff tables, one for each of these stock levels. The constructed pay-off tables show the benefits that each individual obtains from different combinations of individual and aggregated extractions: as individual *i* increases his or her extraction, the respective payoff increases (at a decreasing rate); on the other hand, as aggregate extraction increases, *i*´s payoff decreases. This simulates the social dilemma between individual and collective interests.

**Fig 1:**
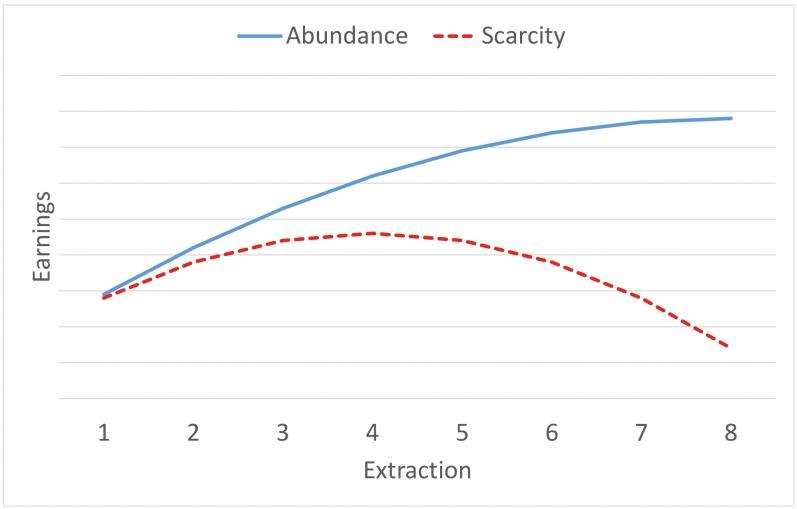
Average payoffs obtained by players under different resource stocks.

Regardless of the abundance of the resource, the socially optimal extraction level is set at one unit. Under an abundant stock, the Nash equilibrium corresponds to an extraction level of 8 units. This corresponds to a corner solution, as the range of plausible extraction is [1, 8]. Under conditions of scarcity, the Nash equilibrium corresponds to an extraction level of 4 units, which corresponds to an interior solution. Notice that although under conditions of resource scarcity the Nash equilibrium is of four units, individuals might still extract any amount between one and eight units. As a result, for this case, players may deviate from the Nash Equilibrium in either a downward or upward direction. Given that cost function is reserve-dependent, the benefits under abundance are higher than those under scarcity; this is true for all levels of extraction. Fig 1 illustrates the average benefits a player may obtain under the two states.

Deviations below the Nash equilibriums imply that individuals incorporate either collective interests into individual decisions, or a consideration of the future consequences of present actions. In the case of low stock, deviations above the Nash equilibrium imply private and social inefficiency, as individuals are making extraction decisions that result in less benefits than those associated with the Nash equilibrium. By doing this, they are increasing the costs for the rest of the fishermen, and they are acting in more resource-harmful manner than theory predicts, thus exacerbating the tragedy of the fisheries.

#### Experimental design

Individuals from the fishing communities were invited to play the games through an open call aimed at adults (18 years or over), preferably those whose main income-generating activity was fishing. No two members from the same household were allowed to be part of the same experimental group. EEGs were performed involving two groups: the first comprised of 230 individuals from eight fishing communities located near the Rosario and San Bernardo Corales National Natural Park (CRSB) in the Colombian Caribbean. The second included 100 individuals from four local communities located close to another marine protected area: the Wildlife Sanctuary of Cienaga Grande de Santa Marta (CGSM), also in the Colombian Caribbean. Descriptive summary statistics for the participants are presented in Table 1. Data collected and surveys used to gather it are available as supplementary material (S1 File).

**Table 1:** Descriptive statistics for participants from both locations. Standard deviations in parentheses.

VARIABLE	CRSB ^a^	CGSM ^b^
**Gender (% women)**	7%	29%
**Age (years)**	35.6 (14.2)	42.1 (15.2)
**Education (years of schooling)**	5.3 (3.4)	4.9 (4.2)
**Main productive activity (% fishers)**	74.5%	55.8%
**Income (USD per month per household)**	282.42 (199.1)	259.47 (268.1)
**Observations**	230	100

^a^ CRSB: Rosario and San Bernardo Coral Reef National Natural Park.

^b^ CGSM: Wildlife Sanctuary of Cienaga Grande de Santa Marta.

For the game, individuals were organized into groups of five participants, and for every round, each player had to privately decide a level of extraction from one to eight units of resource. Individuals were seated back to back in order to guarantee the anonymity and confidentiality of individual decisions.

A supervisor monitored and controlled the game in order to ensure that rules were understood and adhered to. The supervisor was also in charge of collecting the cards on which the participants wrote their extraction decisions and of helping participants to handle material related to the game. Experts on communities’ environmental education explained the game to the fishermen using different visual aids such as drawings, pictures and posters. Three practice rounds were performed before starting the actual game.

In order to guarantee ethics standards, we used the accepted protocols and prepared a written consent that was signed for every participant before starting the activity. All data were analyzed anonymously.

A player’s extraction decisions generate points, which can be converted into monetary units. On average, at the end of the game, US$10 was paid to each person, equivalent to a daily personal wage for the region being considered.

The dynamic part of the game was designed as follows: if in a given round *t* the aggregated extraction (that is, for a five-person group) exceeded 20 units, during the next round (round *t+1*), the group would be faced with resource scarcity. Consequently, during round *t+1*, they would use the low availability payoff table to calculate their earnings. On the other hand, if extraction by the full group during period *t* was less than or equal to 20 units, for period *t+1*, the resource would be abundant; less effort would be required per unit of catch, and the activity would generate higher returns. In that case, the group would use the high availability payoff table in the following round (*t+1*).

For the analysis in this study we considered a phase of ten rounds in which players face the resource under an open access framework (there were no additional management rules).

It is important to remember that, according to the profit maximization model and assuming completely myopic behavior, the expected Nash equilibrium for the game under abundance reflects an individual extraction of 8 units per player. Under scarcity, the Nash equilibrium implies 4 units of extraction per player. The social optimum is a level of extraction of 1 unit per player.

### Methodological approach

In order to analyze the behavior of participants in the EEG, and to address the research question, we adopted the following methodological approach, wherein we used individual extraction decisions from the phase of the game that recreates open-access conditions.

We analyzed the frequency of individual extraction decisions and deviations from the Nash equilibrium at each resource state, and classified those decisions according to their relationship with the theoretically predicted equilibriums. Based on this analysis, we looked for decision patterns that help to explain players’ behavior, particularly when they decide to extract above the Nash equilibrium. Our categories of individual behavior are drawn from this analysis.

As with individual decisions, we searched for group decision patterns, especially when these decisions fall above the Nash equilibriums. We then constructed categories that reflected group behavior.

We examined the relationship between individual behavior and group behavior so as to identify patterns of extraction and the effect that groups can have on individuals.

Finally, we ran an econometric model that explains inefficient extraction decisions (in two ways) as a function of the resource level, the extraction behavior, the type of group and some socioeconomic factors.

## Results

### Individual decisions

**Fig 2:**
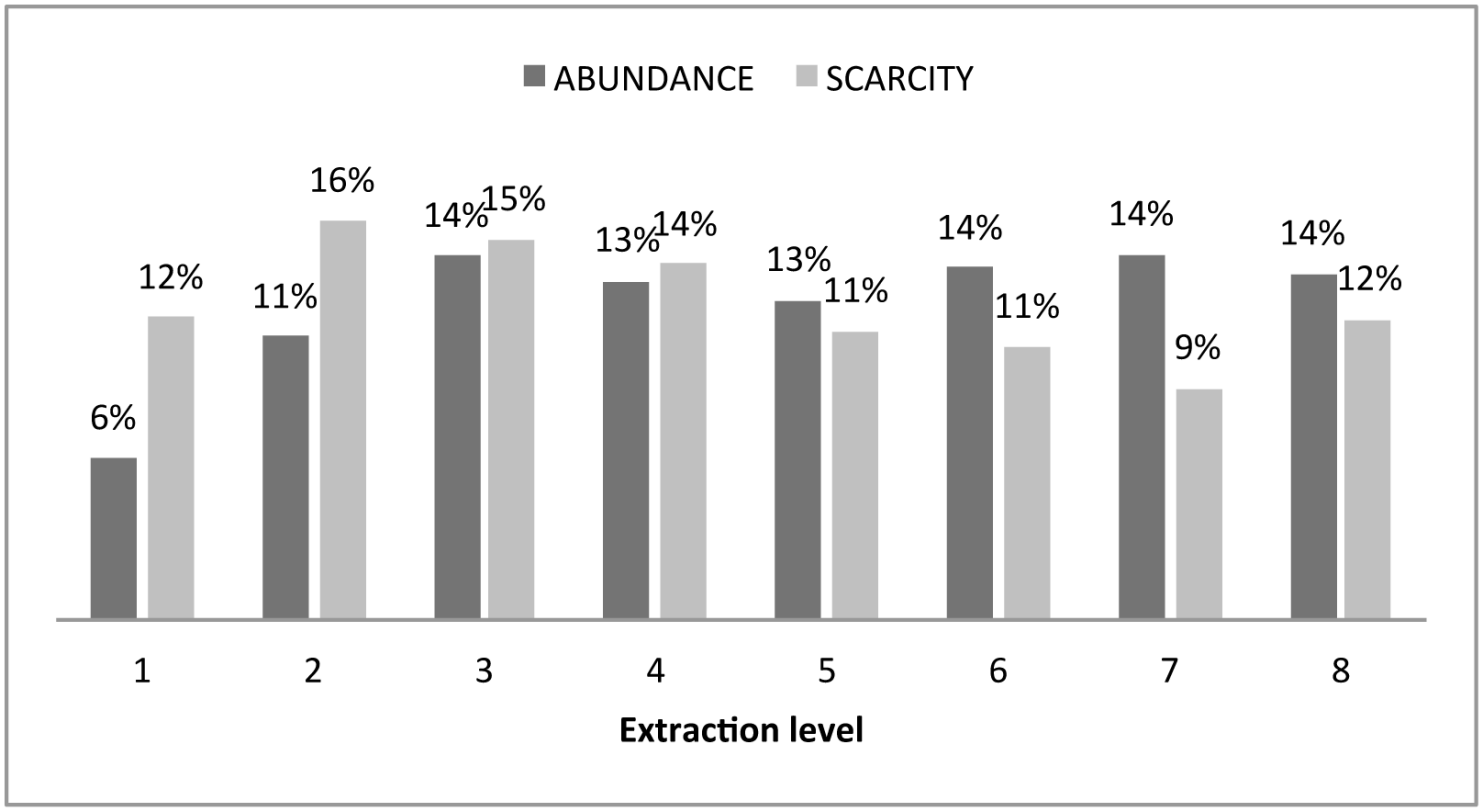
The frequency of different extraction decisions under abundance (high level of stock) and scarcity (low level of stock) of the resource.

Our first step was to analyze the frequency of extraction decisions for every state. Fig 2 shows that when players faced abundance (high stock), 3, 6, 7 and 8 units of extraction were frequent. That is, we observed extraction decisions of more than five units with greater frequency.

When players were confronted with scarcity, extraction distribution was more concentrated across low levels of extraction, with 2 being the most frequent extraction level. Given that the Nash equilibrium for low stock is internal, extractions above four units should not be observed, as they generate lower benefits than those obtained at the level of extraction that is privately efficient. Extractions above four units under scarcity are inefficient, both privately and socially, since the resource is being overexploited without any marginal benefit and, in fact, even at a marginal loss for the individual and for the group. From all the rounds when players faced low stock availability, 43 percent of the decisions were above the Nash equilibrium.

To compare the extraction decisions for the two levels of stock, we calculated the difference between actual extraction and the expected private Nash equilibrium. This measure is what we call the deviation from the Nash equilibrium.

**Fig 3:**
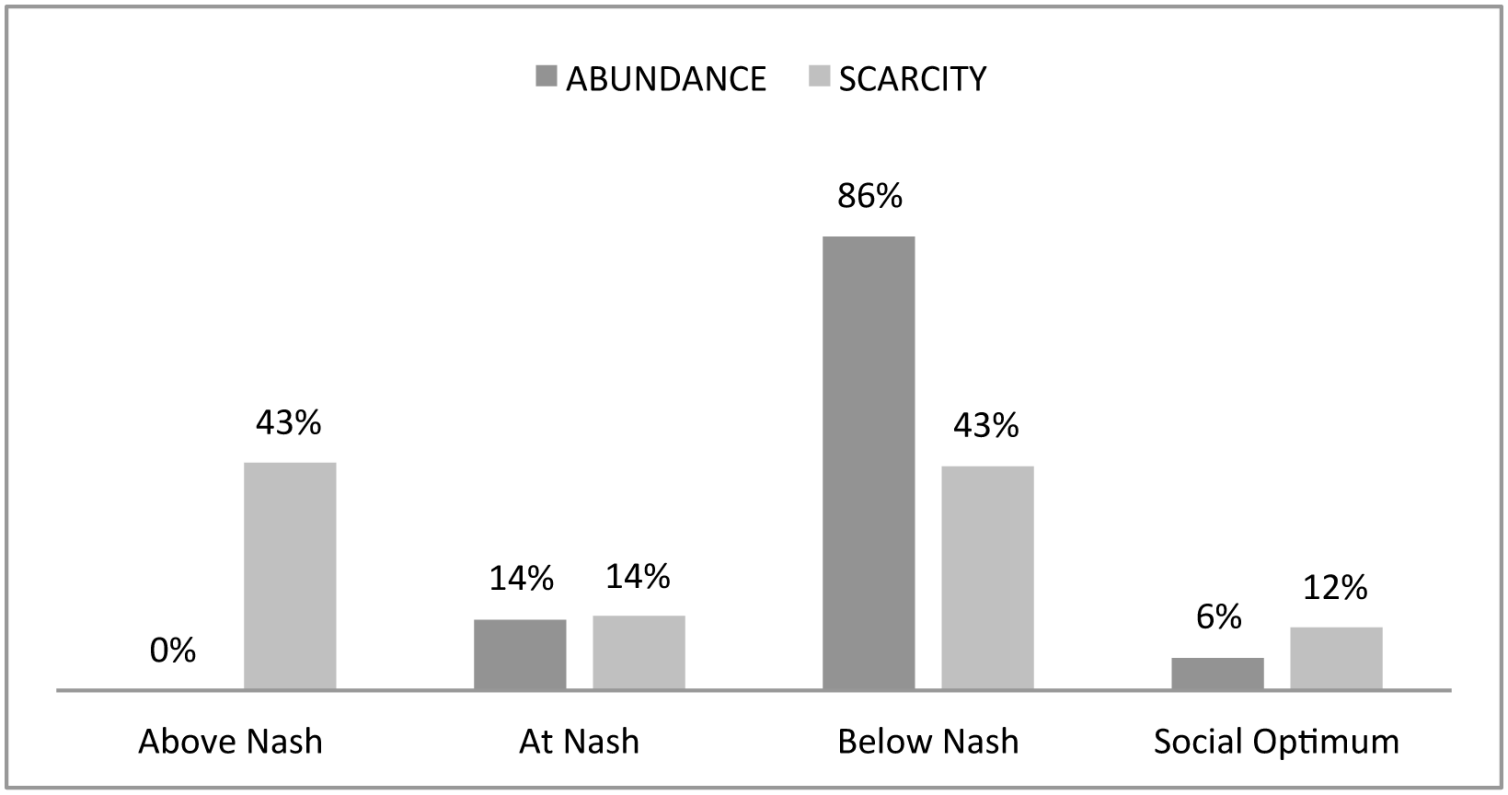
Classification of the deviations from the Nash equilibrium at each resource state.

These deviations are classified in groups according to their respective Nash equilibriums. Fig 3 shows that under high stock (abundance), 86 percent of decisions were below the Nash equilibrium, implying that they reflect either other-regarding preferences or forward-looking behavior. For low stock (scarcity), 86 percent of decisions were made outside of the Nash equilibriums, though 43% were inefficient decisions, as they were made above the Nash equilibrium. It is worth noting that when the Nash equilibrium was closer to the social optimum (under scarcity), players arrived at the social optimum more frequently (12% against 6%) than when Nash equilibrium was set at eight units (under abundance).

In sum, when faced with scarcity, individuals tended to utilize inefficient extraction strategies for almost half of the rounds, thus exacerbating the tragedy of the commons by extracting not only more than the social optimum, but also more than their respective private Nash equilibrium. When scarcity appears, either collective action or forward-looking strategies are reduced. Additionally, the greater the “distance” between the Nash equilibrium and the social optimum, the less likely it is that players will arrive at the optimum.

Our results coincide with those found in some of the economic literature where it is shown that individuals will likely deviate upward or downward from Nash equilibriums, particularly where public goods are concerned [20]. In accordance with those authors we found for the mirror case of a CPR, that when confronted with a situation of scarcity, players will over-extract the resource—that is, they will make decisions above the Nash equilibrium. In doing so, they obtain less profits, undermine the other-regarding interests and exacerbate the tragedy of the commons.

One may think that over-extracting behavior under scarcity may be associated with certain particular individuals, and not with the whole set of players. To test this hypothesis, we divided the sample according to the number of rounds in which every participant played above the Nash equilibrium. Note that this analysis is only possible when players faced scarcity.

**Fig 4:**
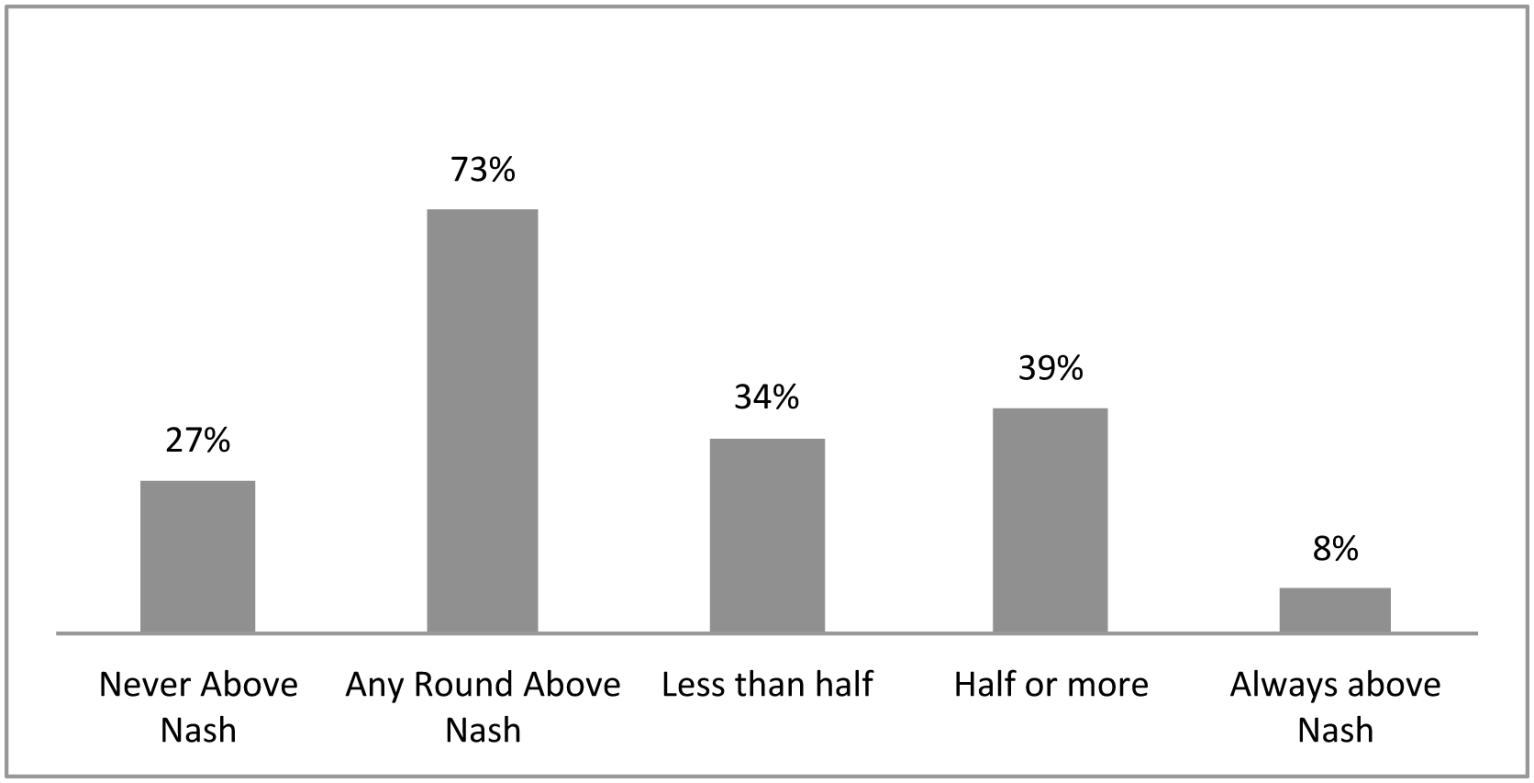
Proportion of individual extraction decisions when facing scarcity classified relative to Nash equilibrium.

Based on this, participants were categorized depending on the average number of rounds in which they decided to play above the Nash equilibrium: some never played above it, others played above it sometimes but in less than half of the rounds, while others played half or more than half of the rounds above it. The results, presented in Fig 4, show that some players never played above the Nash equilibrium; these are known to have pro-social attitudes (27%). Others played less than half of the rounds under scarcity above the Nash equilibrium (although they did so at least once), and these have individualistic attitudes towards Nash equilibrium, although these are skewed to inefficiency (34%). And others have exacerbating attitudes, which are strongly inefficient, even if suffering economic losses as a consequence of acting in this way; these players played above the Nash equilibrium for at least half of the rounds under scarcity (39%). Within this latter group, eight percent of the full sample that faced low stock always played above the Nash equilibrium.

Comparing these results with those of van Soest and Vyrastekova [23], cooperative participants are those that never played above Nash; competitive players, those that play less than half of the rounds above Nash; and individualistic players are those playing half or more of the rounds above Nash. However, they did not consider these possibilities of over-extracting the resource in an inefficient way. When compared with the characterization made by Ostrom [22], it seems evident that group 6 (*always cooperate* as per Ostrom's classification) would match our group of players that never play above the Nash, while group 4 (*never cooperate* as per Ostrom's classification) would be more closely related with those that played around or above the Nash. However, for the other categories it is necessary to consider not only the individual behavior, but also the behavior of the group associated with each player.

### Group decisions

**Fig 5:**
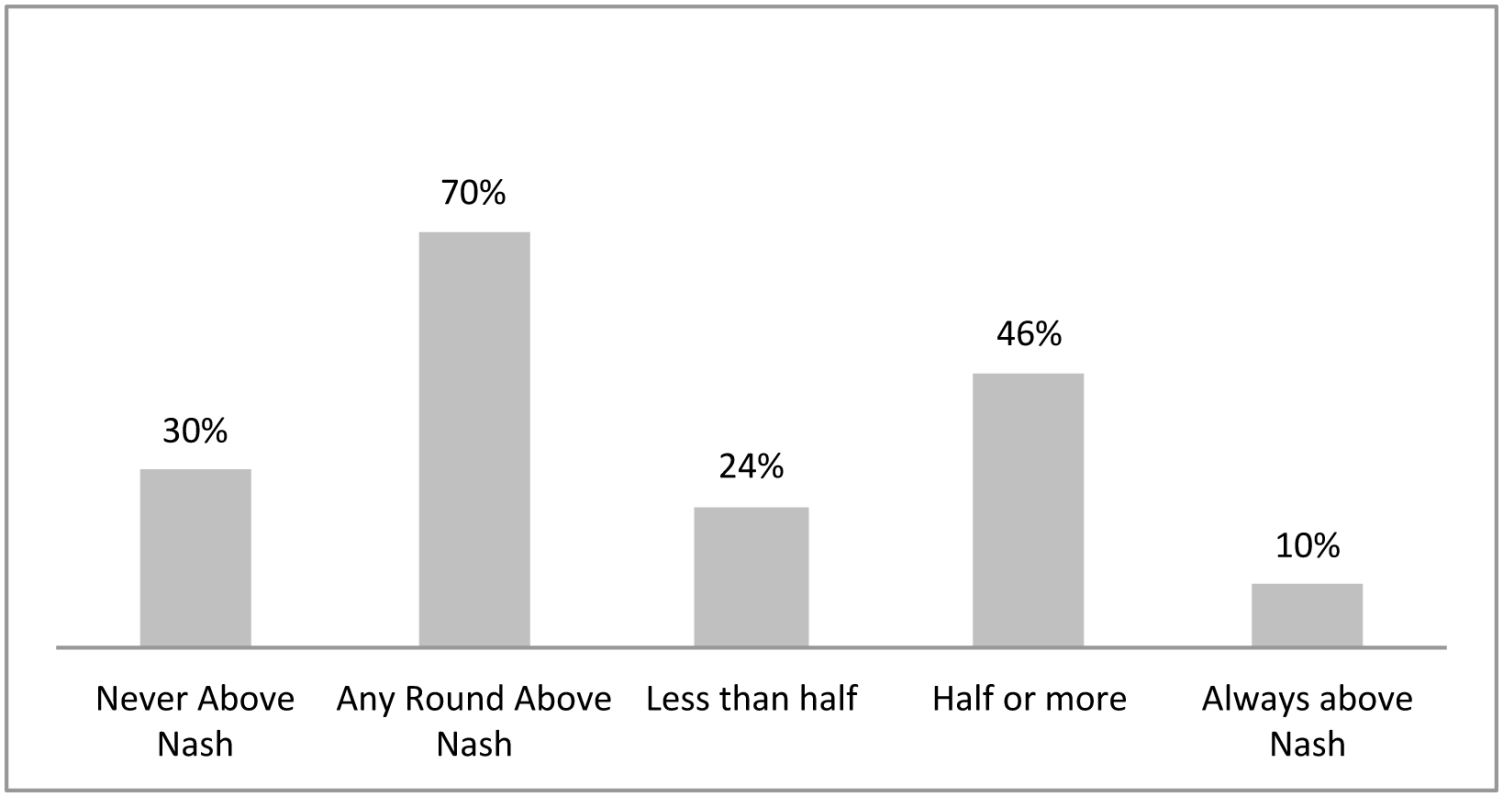
Proportion of group extraction decisions when facing scarcity classified relative to Nash equilibrium.

To analyze group behavior, we performed the same analysis for groups as we did for individuals. As shown in Fig 5, some groups known as “pro-social” groups in social and environmental terms (30%) consistently behaved cooperatively by never extracting above the Nash equilibrium -; some other groups exhibited behavior around the Nash equilibrium (24%); and others still repeatedly extracted in an inefficient way and are known as “exacerbating” groups in social and environmental terms (46%).

**Fig 6:**
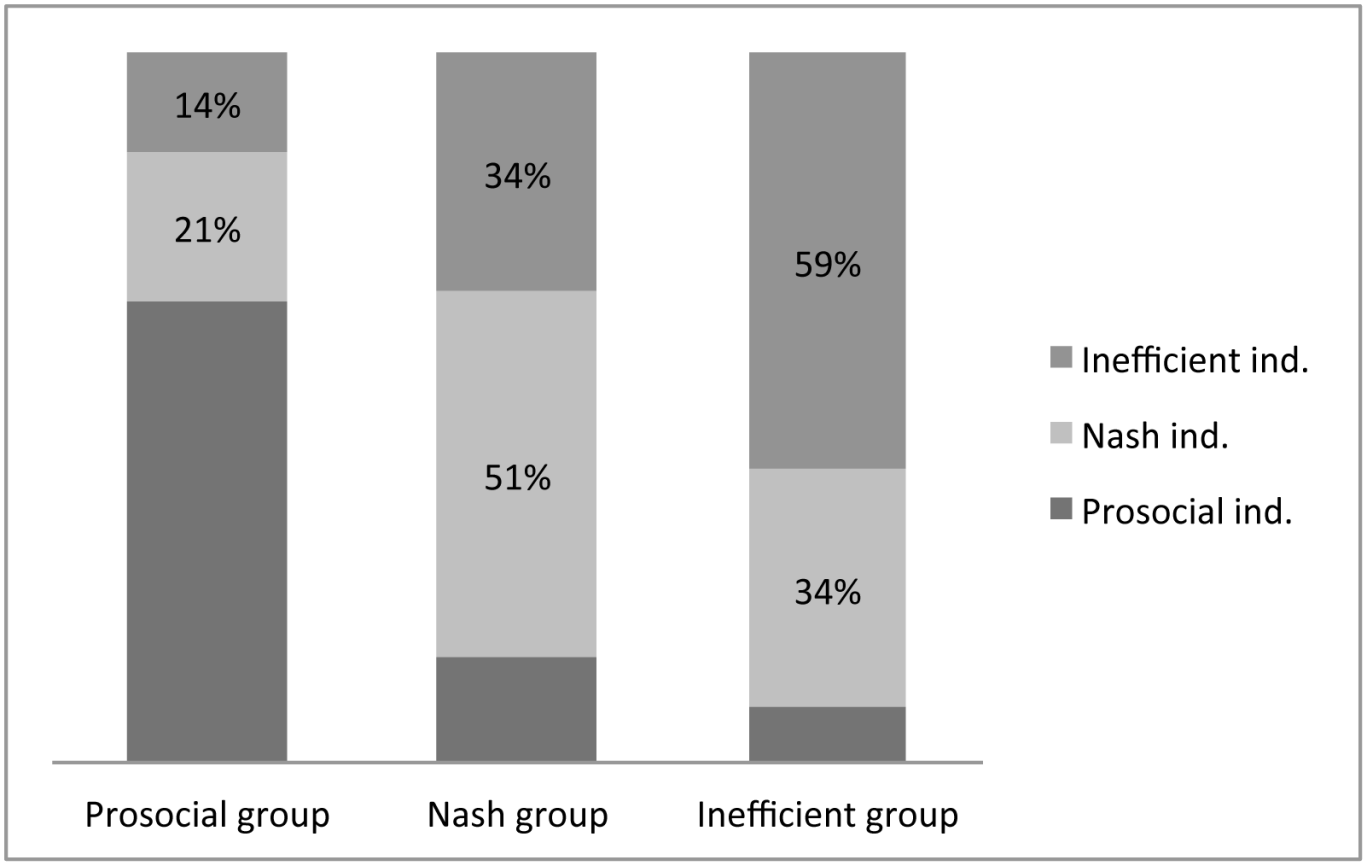
The relationship between individual and group behavior with respect to the Nash equilibrium according to proposed types.

Inasmuch as there are individuals that behave inefficiently throughout the game and individuals that consistently exhibit pro-social behavior throughout the game, (i.e., groups that show similar patterns), the question becomes whether or not “pro-social individuals” always belong to “pro-social” groups. In other words, if peer effect affects decisions. In Fig 6, we observe that social players coincide with social groups for a high proportion of the rounds associated with social groups (65%). Similarly, although not as evident, inefficient individuals exacerbating coincide with exacerbating groups for almost 60% of the rounds.

In those groups that over-extracted the resource, eight percent of the players behaved pro-socially, in the sense that they made an effort to not over-extract the resource. They consistently tried to reduce the group’s extraction, but their effort was canceled out by the inefficient behavior of the rest of the group. As a result, most of the time they were confronted with scarcity and consequently their profits were reduced. Conversely, 14 percent of the players belonging to pro-social groups consistently over-extracted and derived profit from it. At the same time, the pro-social behavior of their respective groups kept them in abundance, as a consequence of which they ended up making greater profits. In essence, they are free riders that took advantage of high levels of extraction while their groups, through their overall efficient and pro-social decisions, maintained high resource availability.

**Fig 7:**
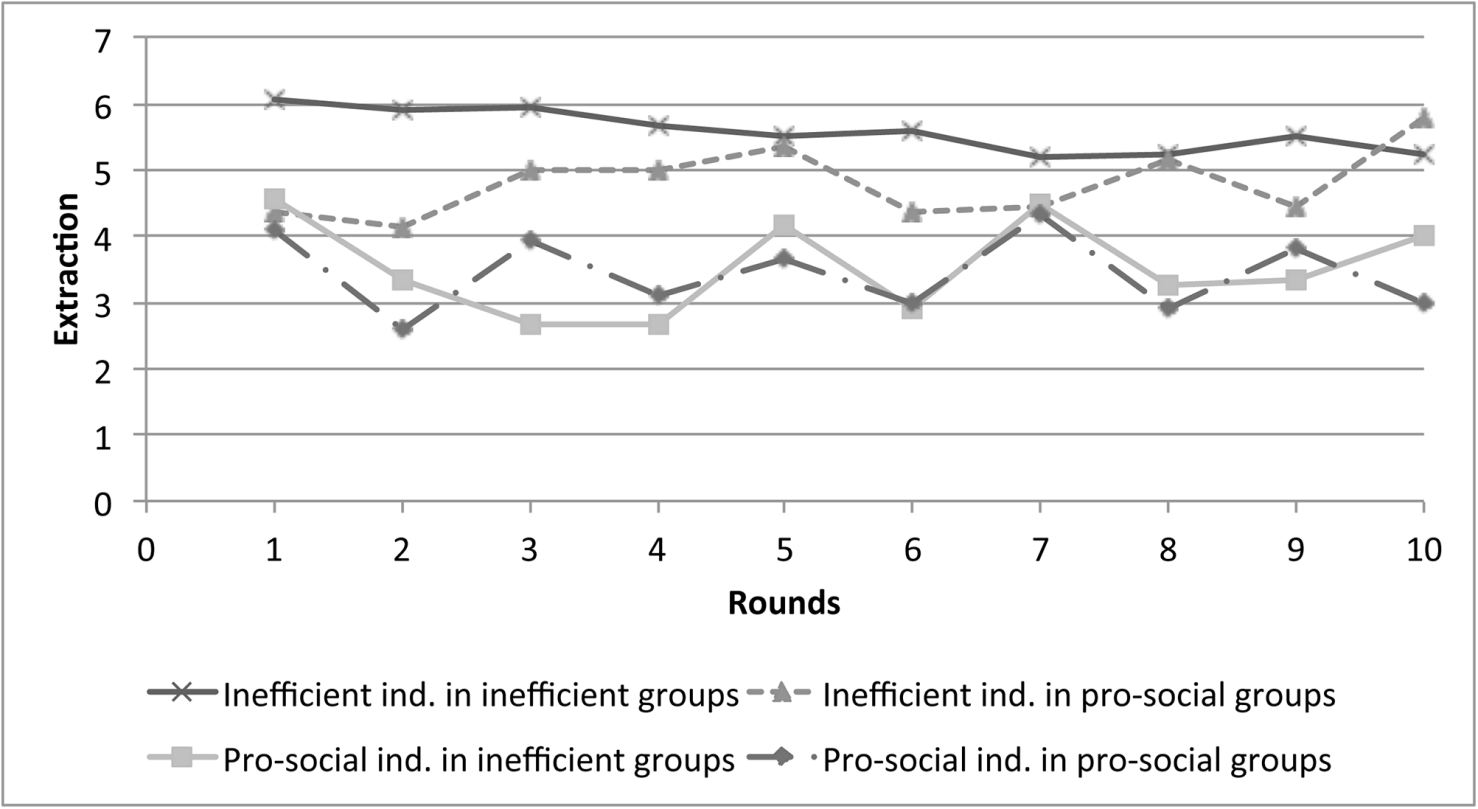
The average extraction of individuals according to proposed categories.

These free riders may erode the pro-social behavior of their group and induce good players to start playing inefficiently. On the other hand, pro-social players in exacerbating groups may send signals to the other players through their behavior that they should reduce their over-extraction. To analyze whether such cases were observed, we calculated the average extraction decisions of players categorized according to their types and the type of group to which they belonged: pro-social individuals playing in either pro-social or inefficient groups, and inefficient individuals playing in either pro-social or inefficient groups. The results are presented in Fig 7. We can see that, in fact, inefficient players maintained inefficient behavior throughout the game as on average they extracted above four units, while the conservative behavior of pro-social individuals was evident as average extraction was consistently lower than four units.

However, the behavior of the inefficient players seems to be influenced by their respective group’s behavior, as their average extraction was lower when belonging to a pro-social group than when being part of an inefficient group (5.6 vs. 4.8 units on average). These two groups would be comparable with group (5) defined by Ostrom [22]. On the other hand, the behavior of the pro-social players does not seem to be affected by the type of group to which they belong; in both cases, the average extraction was of around 3.5 units. That is, pro-social individuals are more inelastic to the peer effect than inefficient participants. In that sense, these sub-groups are comparable with categories (1) and (2) according to the taxonomy presented by Ostrom [22].

### Econometric analysis

So far, we have observed that under conditions of scarcity, individuals tend to exacerbate the tragedy of the commons, and that players can be organized into categories. We now want to formalize these findings through a parametric analysis.

In the proposed econometric model, we might have different expressions of the explained variable. One of them is a dummy that takes the value of one when the individual extracted above the Nash, and zero otherwise. In this case, the model is a binomial probit. Another one is the extraction/Nash-equilibrium ratio, which will be greater than one when the individual is exacerbating the use of the resource. Given that the values of this variable are limited to the interval [0, 2], the specification requires a censored model.

The dependent variable is explained by a set of variables: i) the availability of the resource during the previous round, using a dummy that takes the value of one if the resource was scarce or zero if it was abundant in the previous round; ii) information from the game itself: the individual’s previous extraction (*x*_*i*,*t-1*_), and the aggregated extraction of the rest of the group during the previous round (Σ_-i_ *x*_*t-1*_); iii) the type of group to which a participant belongs, whether it is a pro-social or an inefficient group; and iv) certain demographic characteristics such as education level.

Given that the decisions of each individual over ten rounds are not independent, we adopt a panel data structure so that any error associated with the rounds within a particular player can be separated from errors related to between-individuals variations. As the model uses lagged variables, information about the first round is dropped. The results for the estimated econometric models are presented in Table 2.

**Table 2:** Panel regressions explaining inefficient decisions with respect to Nash equilibrium.

VARIABLES	Model 1: Extract above Nash eq.	Model 2: Extraction/Nash-eq. ratio
**Resource stock scarcity in previous round (1 = scarce, 0 = abundance)**	-0.0858	0.0856***
	(0.0773)	(0.0218)
**Own extraction in previous round (1–8 units)**	0.180***	0.0398***
	(0.0166)	(0.00526)
**Other group-members’ extraction in previous round (4–32 units)**	0.0980***	0.0240***
	(0.00805)	(0.00212)
**Being part of a pro-social group (1 = yes, 0 = no)**	-0.571***	-0.112***
	(0.139)	(0.0427)
**Being part of an inefficient group (1 = yes, 0 = no)**	0.702***	0.213***
	(0.107)	(0.0383)
**Education (years of education)**	-0.0422***	-0.0141***
	(0.0122)	(0.00417)
**Constant**	-3.544***	0.340***
	(0.208)	(0.0554)
**Observations**	2,709	2,709
**Number of groups**	301	301
**Wald chi**	447.44***	460.44***

Standard errors in parentheses

*** p<0.01.

Given the construction of the dependent variables, the relationship with the independent variables should be interpreted as follows: positive coefficients imply that any increase in the independent variable will result in a greater inefficient attitude by the player. Conversely, negative coefficients mean that an increase in the independent variable will result in more pro-social behavior.

The findings show that scarcity generates a positive effect on over-extraction: scarcity of the resource induced players to extract more even if this was inefficient. This result is valid for the second model, and confirms the central hypothesis of this study: scarcity promotes exacerbation of the tragedy of the commons.

On the other hand, every additional unit of individual extraction during the previous round (*t-1*) will result in an increase of over-extraction during the current round, exhibiting some inertia from the players’ decisions. The same effect is observed for the case of extraction from the other members of the group: the more they extracted, the more the individual was incentivized to over-extract the resource. However, the magnitude of the effects is about half of the own effect.

The categorical variables used to capture group effects show significant coefficients and the expected signs: being part of a pro-social group is associated with deviations towards the social optimum, while being in an inefficient group is associated with deviations towards inefficient behavior by the player. These results confirm the conclusion that the type of group matters and that there is a peer effect on decisions that overuse the resource.

Finally, with respect to the level of education, it was consistent with the finding that more educated players tended to move away from inefficient behaviors.

## Conclusions

The objective of this paper was to analyze inefficient extraction behavior in experimental games where scarcity of the resource constrains the possibilities of pro-social behavior and to associate the type of players to such outcomes. To do this, we developed an EEG for a CPR with real fishing communities in the Colombian Caribbean, and simulated two stock levels (scarcity and abundance), which in turn generated two Nash equilibriums.

Although other EEGs performed with local communities have demonstrated that individuals deviate downward from the Nash equilibrium—deviating away from the myopic and individualistic behavior predicted by non-cooperative game theory and investigated by Hardin [6]—our findings show that under scarcity, individuals reduce their pro-social behavior, and might even be privately inefficient, and thus deviate upward from the Nash equilibrium. In terms of resource sustainability, such inefficient behavior implies that individuals not only obtain less profit and undermine others regarding preferences, they also exacerbate the tragedy of the commons.

Much as with previous studies [20, 21], we found—for the case of CPR games—that the “distance” from the theoretical social optimum to the private Nash equilibrium is important for defining the chances of arriving at the social optimum. Under scarcity, the private Nash equilibrium is closer to the social optimum; therefore, we are more likely to observe individuals making decisions that correspond with that optimum.

We also observed that the behavior of players throughout the game exhibited some consistency and therefore they can be classified into groups: some participants maintain their pro-social behavior during the game, regardless of the behavior of the rest of the group, while others maintain their inefficient behavior even if the group is acting pro-socially. As with Isaac and Walker [20], we found that these individuals act as free riders vis-à-vis their fellow group members; they extract at the Nash equilibrium or over it, even while the latter consistently remain “highly cooperative,” and try to deviate downward from the Nash equilibrium.

Groups of players can also be classified into categories: prosocial, Nash-type and inefficient. Similarly to Herr, Gardner and Walker [17], who analyzed the effect of time on externalities associated with the extraction of non-renewable resources, we find that the myopic behavior of individuals not only exacerbates the CPR problem, but also affects the behavior of non-myopic individuals, leading all group members to a race for the resource. Group behavior matters and individual extraction is related with the type of group to which individual belongs, reflecting reciprocity attitudes: “*they extracted more during the previous period therefore I will extract more during the current period*.” In spite of that, prosocial individuals tend to be more inelastic to peer pressures and they maintain their attitudes throughout the game regardless of the behavior of the rest of the group. Inefficient individuals exhibit a more elastic behavior, which is more strongly influenced by the performance of the group.

Our findings provide information that might be useful in the formulation of management strategies for common-pool resources; in particular, when CPRs are facing deterioration and local users perceive them as scarce. Individuals might be less interested in cooperating when the resource is becoming depleted. Therefore, with respect to resources that are highly threatened, management strategies might best focus on zoning, the establishment of non-take zones and the exerting of control. However, when a resource is abundant, management strategies might be more effective if local user participation is involved.

This research shows that the collective behavior of individuals facing a CPR dilemma in an EEG is not a rule, and depends on the condition of the stock; under scarcity, individuals deviate from cooperative behavior and may even engage in a race to the bottom. And, as discussed by Ostrom [22] and van Soest & Vyrastekova [23], individuals come to the game with some given characteristics that are expressed throughout that game and that can be shaped by the performance of the group.

## Supporting Information

S1 File Contains all the data supporting the analysis as well as the surveys used. (ZIP)
