# Supplementary material for: Exacerbating the Tragedy of the Commons: Private Inefficient Outcomes and Peer Effect in Experimental Games with Fishing Communities
Source: PLoS One. 2016 Feb 10;11(2):e0148403. doi: 10.1371/journal.pone.0148403 (PMC4749167; doi:10.1371/journal.pone.0148403)
Supplement: S1 File — (ZIP) [file pone.0148403.s001.zip › data/surveyCGSM.pdf]

ANEXO 6. Encuesta a Jugadores

ENCUESTA INDIVIDUAL REALIZADA A LOS PARTICIPANTES DE LOS JUEGOS ECONÓMICOS EXPERIMENTALES EN LA CGSM

Fecha: ____ ____ ______
DD MM AAAA
Encuestador: ______________________________
Encuesta No: ________

Leer este párrafo antes de empezar con la encuesta para aclarar la confidencialidad de la información: Es importante que usted sepa que la información de estas encuestas será utilizada exclusivamente para fines académicos y de investigación y que las respuestas son absolutamente confidenciales, es decir, que se guardará y se respetará el anonimato de cada una de las personas encuestadas.

1. IDENTIFICACIÓN DEL ENCUESTADO

1.1. Género del Encuestado
Femenino____ Masculino_____

1.2. ¿Cuántos años tiene usted? _______Años

1.3. ¿Nació usted en esta comunidad?
Si: ☐ No: ☐

1.4. ¿Desde hace cuánto tiempo vive su familia y usted en esta comunidad?:
Años: _____ Meses: _____

1.5. Por favor enumere las personas que forman parte de su hogar, es decir, los que comen de la misma olla, y mencione el máximo nivel de educación terminado por usted y por cada uno de ellos.

Relación con usted	Edad	¿Cuál es el último nivel de educación completado?
1. Usted mismo		
2.		
3.		
4.		
5.		
6.		
7.		
8.		
9.		

2. SOBRE LA ACTIVIDAD ECONOMICA DEL ENCUESTADO

1.1. ¿Cuál es su principal actividad económica? ¿Cuál es la actividad económica que le genera la mayor cantidad de ingresos durante el año? **Mencione sólo la MAS IMPORTANTE**:

1. Pesca (pescado, jaiba, camarón, otros) _____
2. Jornalero de fincas agrícolas o ganaderas _____
3. Agricultor en su propio terreno _____
4. Comerciante de pesado, camarón, jaiba o similares ____
5. Agricultor en terreno arrendados _____
6. Construcción _____
7. Servicios de transporte _____
8. Otro, ¿Cuál? ____

1.2. ¿Qué porcentaje de sus ingresos provienen de esa actividad?

Todo ☐ Más de la mitad ☐ La mitad ☐ Menos de la mitad ☐

Si es posible indique en qué porcentaje: ________%

1.3. ¿Hace cuánto tiempo se dedica a esta actividad? _____ Años _____ Meses

1.4. ¿Cuál es su actividad económica secundaria? Mencione otra actividad económica que le genere ingresos durante el año y complemente la actividad mencionada en la pregunta anterior **Puede seleccionar más de una opción**

A. Pesca (pescado, jaiba, camarón, otros) _____
B. Jornalero de fincas agrícolas o ganaderas _____
C. Agricultor en su propio terreno _____
D. Comerciante de pesado, camarón, jaiba o similares ____
E. Agricultor en terreno arrendados _____
F. Construcción _____
G. Servicios de transporte _____
H. Otro, ¿Cuál? ____

1.5. El ingreso de su hogar completo (incluidos todos los miembros) en un mes promedio es:

1. Menor a 200.000 pesos ____
2. Entre 200.001 y 400.000 pesos _____
3. Entre 400.001 y 600.000 pesos ____
4. Entre 600.001 y 800.000 pesos ____
5. Entre 800.001 y 1 millón de pesos____
6. Entre 1.400.001 y 2 millones de pesos ____
7. Entre 2 y 5 millones de pesos _____
8. Más de 5 millones de pesos____

1.6. Suponga que hay una escalera en su municipio/localidad con 10 escalones, en la cual las familias más pobres se encuentran en el primero y las más ricas en el décimo. ¿En cuál escalón se ubicaría usted con su familia? **Mostrar la tarjeta con los escalones y permitir que el encuestado señale el escalón**

**1**	**2**	**3**	**4**	**5**	**6**	**7**	**8**	**9**	**10**

3. SOBRE LA ACTIVIDAD PESQUERA EN LA COMUNIDAD Y ESTADO DE LA CGSM

3.1. En los últimos 10 años ¿Cómo cree usted que ha variado el recurso pesquero? (***Marque con una X)***

1. Ha disminuido		2. Ha aumentado		3. Se mantiene igual	

3.2. En caso que la pesca haya disminuido, ¿Cuáles considera usted son las dos razones más importantes que han generado esta situación? (***Marcar con una X, espere que la persona piense y responda. Si es posible, ubique la respuesta en una de las opciones y marque con un uno la razón principal y con un 2 la razón secundaria. Si el encuestado no responde o no recuerda, mencione las opciones***)

A. Sobre pesca y captura de tallas pequeñas		B. Mayor número de pescadores		C. Expansión de cultivos agrícolas o ganadería	
D. Mayor demanda por pescado		E. Contaminación de ciénaga		F. Restricciones locales de uso	
G. Uso de artes de pesca inadecuadas		H. Sedimentación de la ciénaga		I. Otra razón	

Cúal?____

3.3. ¿Cómo cree usted se deben manejar los recursos pesqueros en la ciénaga?

	**Si**	**No**
Con regulación de las autoridades pesqueras y/o ambientales		
Con regulación de comunidades (normas impuestas por las comunidades)		
Realizando acuerdos entre autoridades y comunidades		
¿Otra forma?		
¿Cuál?		

3.4. ¿Cree usted que las personas de esta comunidad cooperarían para tratar de resolver entre todos, los problemas relacionados con la disminución de la pesca?

Si: ☐ No: ☐

3.5. Cuáles cree usted que son los DOS problemas más importantes que más afectan la CGSM? Escriba primero el más importante.

1. ____
2. ____

3.6. A partir de su experiencia y su conocimiento de la zona, cuáles serían las mejores maneras de resolver estos problemas:

1. ____
2. ____
3. ____

3.7. ¿Qué es lo que usted más valora de la Ciénaga Grande de Santa Marta?

____
____
____
____
____

4. CAPITAL SOCIAL

4.1. ¿Usted hace parte de alguna organización comunitaria? **En caso negativo pase a la pregunta 4.4**

Si: ☐ No: ☐

4.2. ¿A cuál organización pertenece?

____
____
____

4.3. ¿Cuál es la razón principal que lo llevo a usted a asociarse? **(Marcar con una X, espere que la persona piense y responda. Si el encuestado no logra responder, mencione las opciones. Marque solamente una, la más importante para el encuestado)**

1. Mejores precios	☐	2. Obligado o presionado por el gobierno, líderes, amigos o familiares	☐
3. Conservación de los recursos en la CGSM	☐	4. Aspectos sociales (hacer amigos, trabajar en conjunto con otros pescadores, temor de ser excluido, etc.)	☐
5. Apoyos en épocas de bajo ingreso	☐	6. Razones asociadas con el conflicto en la región	☐
7. Ser respetado y reconocido como miembro de la organización	☐	8. Mejorar comercialización	☐
9. Otros	☐	Cuales ______________________	☐

4.4. ¿Por qué razón no hace parte de ninguna organización? **Puede mencionar más de una opción**

A. Ya ha hecho parte de alguna (s) en el pasado y fracasó ______
B. La gente en la comunidad es individualista _______
C. A la gente de la comunidad no le gusta trabajar en equipo ______
D. No es fácil confiar en los demás ______
E. No le gustan las organizaciones comunitarias ______
F. Otra razón_____
¿Cuál?
____
____
____

4.5. ¿Cree usted que los recursos asociados a la Ciénaga son _______________ para el bienestar de sus hijos o nietos?

1. Muy importantes
2. Importantes
3. Poco importantes
4. Nada importantes

4.6. Usted cree que la gente en esta comunidad es:

1. Siempre confiable __________
2. Algunas veces confiables_______
3. Pocas veces confiable_______
4. Nunca es confiable______

4.7. Si usted tuviera un problema y necesitara ayuda, usted cree que la gente en esta comunidad:

1. Siempre lo ayudaría __________
2. Algunas veces lo ayudaría_______
3. Pocas veces lo ayudaría_______
4. Nunca lo ayudaría______

4.8. Usted cree que las personas de esta comunidad:

1. Se preocupan solamente por el bienestar propio ___________
2. Se preocupan solamente por el bienestar de su propia familia__________
3. Se preocupa por el bienestar de sus vecinos y amigos_________
4. Se preocupa por el bienestar de TODA la comunidad________

MUCHAS GRACIAS POR SU TIEMPO
