# Supplementary material for: Exacerbating the Tragedy of the Commons: Private Inefficient Outcomes and Peer Effect in Experimental Games with Fishing Communities
Source: PLoS One. 2016 Feb 10;11(2):e0148403. doi: 10.1371/journal.pone.0148403 (PMC4749167; doi:10.1371/journal.pone.0148403)
Supplement: S1 File — (ZIP) [file pone.0148403.s001.zip › data/surveyCRSB.pdf]

Encuesta individual para jugadores

Fecha: ____________________ Grupo ______ Número de jugador ______

Lugar: ____________________

A.1. Cuantos años ha vivido usted en esta comunidad? _____ años

A.2. Por cuanto tiempo sus ancestros (padres, abuelos, etc.) han vivido en esta Isla o Comunidad? ____________ años

A.3. Cual es la actividad económica a la que dedica mayor parte de su tiempo en el año?

Pesca de superficie__ Pesca de buceo __ Fabricación de Artesanías __
Venta de Artesanías __ Agricultor __ Comerciante __
Otro, Cual _________________________

A.4. Cuantas horas a la semana gasta en esa actividad? ___________ horas a la semana

A.5. Cual es la actividad económica que le genera la mayor cantidad de ingreso durante el año?
Pesca de superficie __ Pesca de buceo __ Fabricación de Artesanías __
Venta de Artesanías __ Agricultor __ Comerciante __
Otro, Cual _________________________

A.6. Que porcentaje de sus ingresos provienen de esa actividad? _______ %

A.7. Por favor liste a las personas que viven en su hogar, empezando por usted:

**Relación con usted**	**Edad**	**Sexo**		**máximo nivel de educación terminado**	**Estudia actualmente?**	
1. Usted mismo		M	F		Si	No
2.		M	F		Si	No
3.		M	F		Si	No
4.		M	F		Si	No
5.		M	F		Si	No
6.		M	F		Si	No
7.		M	F		Si	No
8.		M	F		Si	No
9.		M	F		Si	No

A.8. El ingreso de su hogar completo (incluidos todos los miembros) en un mes promedio es:

Menor a 400,000 pesos __
Entre 400,000 y 800,000 pesos __
Entre 800,000 y 1,200,000 pesos __
Entre 1,200,000 y 1,600,000 __
Entre 1,600,000 y 2 millones __
Entre 2 y 5 millones de pesos __
Más de 5 millones de pesos__

B.1. Por favor diga si usted está de acuerdo o desacuerdo con las siguientes afirmaciones, de acuerdo a la siguiente escala:

1 = Totalmente de acuerdo
2 = De acuerdo
3 = En desacuerdo
4 = Totalmente en desacuerdo

**Afirmación o idea**	**Calificación**
1. La mayoría de la gente en esta comunidad es honesta y confiable	
2. La gente en esta comunidad se preocupa más por su propio bienestar	
3. Los miembros de esta comunidad son más confiables que los de otras islas	
4. En esta comunidad uno debe estar alerta o si no, alguien se aprovecha	
5. Si usted tiene un problema, siempre hay alguien de la comunidad listo a ayudarle	
6. Usted no pone atención a las opiniones de otros en la comunidad	
7. La mayoría de personas en esta comunidad están dispuestas a ayudarlo si usted lo necesita	
8. Esta comunidad ha prosperado en los últimos cinco años	
9. Usted se siente aceptado como miembro de esta comunidad	
10. Si a usted se le pierde algo, alguien en la comunidad le ayudaría a encontrarlo y se lo devolvería	

B.2. Si hay un problema relacionado con el uso de los recursos naturales en esta comunidad (por ejemplo, la pesca), cree usted que la gente cooperaría para tratar de resolver el problema?

Con toda seguridad __
Probablemente __
Difícilmente __
Muy difícilmente __

B.3. Durante el último año, se ha reunido usted con otras personas de la comunidad para tratar de resolver un problema relacionado con recursos de uso común, como la pesca o la extracción de los corales:

Si ____ No ____ Cuantas veces? __________

B.4. Conoce usted a algún líder o alguna organización que represente a su comunidad ante las autoridades?

Si ____ No ____ Quien es? ______________________________

C.1. ¿Que tan importante considera usted que son los manglares?
Muy Importantes__ Importantes__ Poco importantes__ No importantes__

C.2. ¿Que tan importante considera usted que son los corales?
Muy Importantes__ Importantes__ Poco importantes__ No importantes__

C.3. ¿Que tan importante considera usted que son las playas?
Muy Importantes__ Importantes__ Poco importantes__ No importantes__

C.4. ¿Cree usted que los corales, los manglares, las playas son importantes para sus hijos o nietos? Si__ No__

C.5. Sabe usted que la zona marítima alrededor de las Islas hace parte del Parque Nacional Natural Corales del Rosario y San Bernardo, el cual está protegido por el estado? Si___ No___

C.6. Usted o alguien de su familia ha participado en trabajo voluntario relacionado con el manejo, la conservación o el monitoreo de los recursos naturales en el parque durante el ultimo año?

Si ____ Cuantos días por año? ____
No ___

C.7. ¿Ha recibido algún tipo de información respecto al manejo de los recursos marinos del Parque Nacional Natural? Si__ No__

C.8. ¿Esa información que ha recibido ha aumentado su conciencia o preocupación por la preservación de los recursos naturales?

Mucho __
Algo __
No mucho __
Nada __

C.9. ¿Sabe usted que existen normas en el parque que regulan el uso de los recursos naturales?
Si __ No__

C.10. ¿Cree usted que vale la pena aplicar esta reglamentación? Si__ No___

C.11. ¿Sabe usted que autoridad es la encargada de hacer cumplir estas reglas?
Si__ No__ Cuál? __________________

C.12. ¿Cree usted que aumentar la regulación, las sanciones y la vigilancia es útil para lograr una pesca más sostenible? Si __ No __

C.13. ¿Cree usted que esta regulación la debe hacer una Autoridad gubernamental o la comunidad? Autoridad __ Comunidad __

D.1. ¿Ha sido invitado por alguna autoridad oficial a asistir a reuniones públicas, talleres o cursos para tratar el tema del manejo del parque? Si__ No__

D.2. ¿Las reuniones o talleres con la comunidad son programadas frecuentemente? Si__ No__

D.3. ¿Usted cree que participar en estas reuniones genera beneficios o permite solucionar conflictos relacionados con el uso de los recursos naturales? Si__ No__

D.4. ¿Cree usted que la información recibida en estas reuniones han generado o pueden generar un cambio en las prácticas usadas por la comunidad? Si__ No__

D.5. ¿Usted cree que la experiencia de pescadores de la comunidad es tenida en cuenta por las autoridades encargadas del manejo del parque? Si__ No__

D.6. ¿Cree que su opinión es tenida en cuenta en las decisiones de manejo del parque?
Si__ No__

D.7. ¿Usted denuncia cuando observa actividades que violan las reglas del parque?
Si__ No__

D.8. ¿Usted cree que vale la pena denunciar? Si___ No___

D.9. ¿Usted piensa que la colaboración mediante denuncias es vista de manera positiva por parte de la comunidad? Si___ No___

D.10. ¿Ha hecho algún tipo de petición a las autoridades del Parque? Si__ No___

D.11. ¿Esta ha sido atendida por las autoridades? Si__ No__

D.12. ¿Usted piensa que la autoridad del parque tiene la capacidad suficiente para hacer cumplir la reglamentación en toda el área del parque? Si__ No__

E.1. ¿Desde su perspectiva, cuáles son los tres problemas que más afectan al parque?, escriba primero el más importante.

1. ______________________________
2. ______________________________
3. ______________________________

E.2. A partir de su experiencia, cuáles serían las mejores maneras de resolver estos problemas:

1. ______________________________
2. ______________________________
3. ______________________________

E.3. Si se propusiera la posibilidad que la gente pudiera contribuir en el manejo del parque, compartiendo responsabilidades con la Unidad de Parques, estaría usted dispuesto a participar?

Si___ No___

E.4. Como cree usted que podría ser su participación? (Marque todas las que crea)

Vigilancia ___
Trabajo voluntario ___
Reducción de la pesca ___
Cumplimiento de las normas ___
Contribuyendo a la definición de las normas para el Parque ___

E.5. Cómo percibe usted el hecho de que esta zona sea un Parque Nacional Natural protegido por el Estado?

Excelente ___
Muy Buena ___
Buena ___
Regular ___
Mala ___
Muy mala ___
Pésima___

F.1. Que tan satisfecho queda con las ganancias del experimento?

Muy satisfecho __
Satisfecho __
Ni lo uno ni lo otro __
Molesto __
Muy molesto __

F.2. Se fijó en el comportamiento de los otros participantes? Si __ No __

F.3. Cambiaron sus decisiones por el comportamiento de los otros? Si __ No __

F.4. Cree que este experimento representa de una forma sencilla las experiencias que usted debe enfrentar en esta comunidad?

Para nada __
Algo __
Más o menos __
Mucho __
Totalmente __

Muchas gracias por su colaboración!
